# Development and Validation of a Clinical Risk Score for Predicting Postpartum Hemorrhage in Women with Antepartum Hemorrhage

**DOI:** 10.34763/jmotherandchild.20263001.d-25-00041

**Published:** 2026-04-30

**Authors:** Swati Asati, Pranshi Asati, Anubhav Gupta, Meenakshi Gothwal

**Affiliations:** Department of Obstetrics & Gynecology, Vyas Medical College & Hospital, Jodhpur, Rajasthan, India; Department of Obstetrics & Gynecology, Autonomous State Medical College Lalitpur, UP, India; Department of Transfusion Medicine, All India Institute of Medical Sciences, Jodhpur, Rajasthan, India; Department of Obstetrics & Gynecology, All India Institute of Medical Sciences AIIMS, Jodhpur, Rajasthan, India

**Keywords:** postpartum hemorrhage, antepartum hemorrhage, risk prediction model, risk score, logistic regression, bootstrap validation, TRIPOD

## Abstract

**Background:**

Postpartum hemorrhage (PPH) remains a leading cause of maternal morbidity and mortality globally, particularly in women with antepartum hemorrhage (APH). Current risk assessment methods lack standardized predictive tools that are both simple and reliable for clinical application.

**Material and methods:**

We conducted a secondary analysis of a prospectively collected cohort of 100 pregnant women presenting with APH at ≥28 weeks’ gestation at a tertiary care centre in northern India. Multivariable logistic regression was used to identify significant predictors of PPH. A point-based clinical risk score was then developed based on the multivariable model and internally validated using bootstrap techniques with 1000 replicates.

**Results:**

PPH occurred in 30% of patients (n=30). Multivariable analysis identified four independent predictors of PPH: maternal age (adjusted odds ratio [OR] 1.29 per year; 95% confidence interval [CI] 1.10–1.51; p=0.002), gravidity (OR 2.11 per unit; 95% CI 1.00–4.43; p=0.049), gestational age at delivery (OR 0.64 per week; 95% CI 0.44–0.94; p=0.021), and antepartum blood transfusion (OR 2.44; 95% CI 1.02–5.84; p=0.045). The prediction model demonstrated excellent discrimination with an area under the receiver operating characteristic (ROC) curve of 0.86 (95% CI 0.80–0.92) and good calibration (slope 0.95). Bootstrap internal validation yielded an optimism-corrected AUC of 0.84. The resulting four-factor risk score stratified patients into four risk categories with PPH rates ranging from 4% (low risk) to 100% (very high risk).

**Conclusion:**

The four-variable score provides an accurate, easily applicable tool with excellent predictive performance. The score is a promising tool that, pending external validation, may facilitate early identification of high-risk patients and improve maternal outcomes. Further research should focus on external validation of this tool in diverse populations and its integration into clinical practice.

## Introduction

Antepartum hemorrhage (APH), defined as bleeding from the genital tract after 28 weeks of gestation, complicates approximately 2–5% of pregnancies and remains a major contributor to maternal and perinatal morbidity worldwide [1,2]. Despite advances in obstetric care, APH continues to present significant diagnostic and management challenges, particularly in resource-limited settings where timely interventions may be constrained. Among the severe complications associated with APH, postpartum hemorrhage (PPH) is a critical concern due to its potential for rapid hemodynamic deterioration and maternal death. PPH is commonly defined by excessive blood loss (≥500 mL after a vaginal birth or ≥1000 mL after a cesarean birth, or clinically significant bleeding requiring transfusion) and occurs in up to 30% of women with preceding APH [3,4]. The relationship between APH and subsequent PPH reflects overlapping pathophysiologic mechanisms including placental abnormalities, coagulopathy, and uterine dysfunction.

Current clinical assessments of PPH risk in APH patients remain largely empirical, lacking standardized predictive tools that combine simplicity with reliability [1,2]. While several studies have examined PPH predictors in general obstetric populations, few have focused specifically on women with APH, where overlapping risk factors and distinct pathophysiologic mechanisms warrant separate consideration [3,4]. Recent advances in predictive modeling —particularly the use of quantitative blood loss measurement and robust internal validation techniques — have enhanced the reliability of obstetric risk prediction tools [5,6]. The development of validated clinical prediction models following established guidelines such as TRIPOD (Transparent Reporting of a Multivariable Prediction Model for Individual Prognosis or Diagnosis) ensures methodological rigor and facilitates clinical adoption [7,8].

**Objective:** This study aimed to develop and internally validate a multivariable logistic regression-based clinical prediction model for PPH in women presenting with APH, translate the model into a practical point-based risk score, and evaluate its discrimination, calibration, and clinical utility using recommended best practices in prediction model research.

## Material and methods

### Study Design and Setting

This study is a secondary analysis of a prospectively collected observational cohort of pregnant women with APH at or beyond 28 weeks’ gestation. The study was conducted at a tertiary care referral center in northern India. Institutional ethics committee approval was obtained, and written informed consent was provided by all participants.

### Study Population and Inclusion Criteria

Consecutive patients presenting with APH due to any etiology (including placenta previa, placental abruption, and unexplained third-trimester bleeding) were included. Inclusion criteria were: singleton pregnancy, gestational age ≥28 weeks, and APH sufficient to require hospital admission. Exclusion criteria included multiple gestation (twin or higher-order pregnancies), known coagulopathy or bleeding diathesis, and incomplete delivery or postpartum records. By design, no patients with multiple gestation were enrolled, and all analyses pertain to singleton pregnancies.

### Primary Outcome Definition

The primary outcome was postpartum hemorrhage (PPH). For this study, PPH was defined as an estimated blood loss ≥500 mL after vaginal delivery or ≥1000 mL after cesarean section, or any amount of blood loss sufficient to require a blood transfusion within 24 hours postpartum. Blood loss was assessed using quantitative techniques wherever feasible, including the use of calibrated obstetric drapes and gravimetric measurement, in accordance with current recommendations for objective measurement of obstetric hemorrhage. This definition combines the traditional volume-based criteria with a clinical criterion (need for transfusion) to capture clinically significant hemorrhage.

### Predictor Variables

Based on literature review and expert clinical opinion, we evaluated eight candidate predictor variables for potential association with PPH. These variables, recorded at admission or during the intrapartum course, included: maternal age (years, treated as a continuous linear variable), gravidity (count of all pregnancies, treated as an ordinal predictor), hemoglobin level at admission (g/dL, continuous), gestational age at delivery (weeks, continuous), number of antenatal care visits (<3 vs ≥3, binary), time from onset of bleeding symptoms to hospital admission (hours, continuous), time from hospital admission to delivery (hours, continuous), and the occurrence of any antepartum blood transfusion prior to delivery (yes/no). These predictors were chosen to encompass maternal baseline characteristics, pregnancy-related factors, healthcare access factors, and proxies for severity of APH. All predictor data were obtained from the hospital records and were complete for the cohort (no imputation was required).

### Statistical Analysis and Model Development

We first compared baseline characteristics between those who did and did not develop PPH, using appropriate univariate tests (Student’s *t* test or Mann-Whitney *U* for continuous variables; chi-square or Fisher’s exact test for categorical variables). A multivariable logistic regression model was then built to identify independent predictors of PPH. All eight candidate variables were entered into the initial model. Given the limited sample size, we minimized model complexity; variables that did not retain significance were removed in a stepwise manner to arrive at a more parsimonious final model. Variables with *p*<0.05 in the final multivariable model were considered significant independent predictors. Model coefficients were estimated with maximum likelihood methods, and results are presented as adjusted odds ratios (OR) with 95% confidence intervals (CI). Model assumptions were checked including linearity of continuous predictors (maternal age, gestational age) and absence of high multicollinearity. Goodness-of-fit was assessed with the Hosmer-Lemeshow test.

**Model performance** was evaluated on several metrics. Discrimination was quantified by the area under the ROC curve (AUC) with 95% CI. Calibration was assessed by examining the calibration slope and intercept (ideal values 1.0 and 0, respectively) and by visual inspection of a calibration plot (observed vs. predicted risk). Overall performance was summarized with Nagelkerke’s R^2^ and the Brier score for predictive accuracy. All performance metrics are reported for the original model (apparent performance).

### Internal Validation

We performed internal validation using bootstrap resampling with 1,000 iterations to correct for potential overfitting. In each bootstrap sample, the full modeling procedure was repeated, and model performance was evaluated. The optimism (the performance decrement observed when applying the model to bootstrap resamples versus the original data) was calculated for the AUC and other metrics. An optimism-corrected AUC was obtained by subtracting the average optimism from the apparent AUC. We also calculated a uniform shrinkage factor to adjust the model’s regression coefficients if needed; this factor is derived from the slope of the linear predictor in the bootstrap validation and indicates the degree of overfitting. Predictor inclusion frequencies across the bootstrap samples were examined to gauge the stability of variable selection.

### Clinical Risk Score Development

After identifying the independent predictors, we translated the final logistic regression model into a point-based clinical risk score for ease of bedside application. Each predictor’s regression coefficient was scaled and rounded to an integer point value, using established methods (e.g., multiplying coefficients by a constant factor and rounding) to ensure the score reflected the relative contribution of each variable. The point assignments were chosen to be clinically intuitive. **Maternal age** was scored as 1 point per 4-year increment above 25 years of age (capped at 2 points maximum). **Gravidity** was scored as 1 point per pregnancy beyond the first (capped at 4 points). **Gestational age** at delivery was scored inversely: earlier gestational ages carry higher risk points (0 points for term weeks; 1 point for 33–36 weeks; and **2 points for 28–32 weeks**. Because the study included only women at ≥28 weeks’ gestation, this category represents the highest risk of prematurity within our cohort). **Antepartum blood transfusion** (indicating a severe APH) was assigned 2 points if a transfusion was given before delivery. The total risk score was the sum of these component points, ranging from 0 (no risk factors) to a maximum of 11 points in this cohort. We defined pragmatic risk categories based on the total score: low risk (0–2 points), moderate risk (3–5 points), high risk (6–8 points), and very high risk (≥9 points). These categories were pre-specified to correspond to roughly quartile divisions of the score distribution and to facilitate clinical interpretation. All statistical analyses were conducted using Python (v3.11) with appropriate libraries for data analysis. The study followed TRIPOD guidelines for transparent reporting of prediction model development and validation [7,8].

## Results

### Baseline Characteristics

Among the 100 women with APH included, 30 (30.0%) went on to develop PPH. Table 1 presents the baseline clinical characteristics stratified by PPH outcome. The mean maternal age of the cohort was 29.3 ± 4.5 years. Women who experienced PPH were on average older (mean 31.0 ± 3.8 vs. 28.5 ± 4.6 years for those without PPH, *p*<0.001). Median gravidity was 3 (interquartile range [IQR] 2–4); patients with PPH had a higher gravidity (median 4 vs. 2 for no PPH, *p*=0.008). The mean hemoglobin level at admission was 9.5 ± 1.3 g/dL, with no significant difference between the PPH and non-PPH groups (both ~9.5 g/dL on average, *p*=0.991). The mean gestational age at delivery was 35.2 ± 2.1 weeks; women who had PPH delivered significantly earlier on average (33.9 ± 1.8 weeks) than those who did not (35.8 ± 2.0 weeks, *p*<0.001). Consequently, 70.0% of PPH cases were in preterm deliveries (<37 weeks) compared to 50.0% preterm among those without PPH (this difference approached significance, *p*=0.067). There was no significant difference in the number of antenatal care visits (e.g., 33% vs. 40% had <3 antenatal visits in PPH vs. no PPH, *p*=0.513). Notably, a much higher proportion of women who developed PPH had received an antepartum blood transfusion prior to delivery (76.7% vs. 12.9% in those without PPH, *p*<0.001), underlining that a severe antepartum hemorrhagic event often preceded PPH. The time from onset of bleeding symptoms to hospital admission and the time from admission to delivery did not differ significantly between groups (mean ~5–6 hours in both, *p*>0.1). These findings highlight that older age, higher gravidity, earlier gestational age, and severe APH (requiring transfusion) were associated with higher incidence of PPH, whereas baseline hemoglobin and timing factors were not clearly predictive (Table 1).

**Table 1.** Baseline Characteristics of the Study Population Stratified by PPH Outcome (N=100) (Values are given as mean ± SD, median (IQR), or number (%) as appropriate).

**Characteristic**	**Overall (N=100)**	**PPH Yes (n=30)**	**PPH No (n=70)**	***P*-value**
**Maternal factors**				
Age (years) – mean ± SD	29.3 ± 4.5	31.0 ± 3.8	28.5 ± 4.6	<0.001
Gravidity – median (IQR)	3 (2–4)	4 (3–5)	2 (2–3)	0.008
Hemoglobin at admission (g/dL) – mean ± SD	9.5 ± 1.3	9.5 ± 1.5	9.5 ± 1.2	0.991
**Pregnancy factors**				
Gestational age at delivery (weeks) – mean ± SD	35.2 ± 2.1	33.9 ± 1.8	35.8 ± 2.0	<0.001
Preterm delivery (<37 weeks) – n (%)	56 (56.0)	21 (70.0)	35 (50.0)	0.067
**Care factors**				
<3 antenatal care visits – n (%)	38 (38.0)	10 (33.3)	28 (40.0)	0.513
Antepartum blood transfusion – n (%)	32 (32.0)	23 (76.7)	9 (12.9)	<0.001
**Timing factors**				
Time from onset to admission (hours) – mean ± SD	5.2 ± 2.8	5.8 ± 3.1	4.9 ± 2.6	0.189
Time from admission to delivery (hours) – mean ± SD	6.4 ± 3.2	6.8 ± 3.5	6.2 ± 3.0	0.438

PPH = postpartum hemorrhage; SD = standard deviation; IQR = interquartile range.

### Multivariable Analysis of PPH Predictors

In the multivariable logistic regression analysis, four variables emerged as independent predictors of PPH (Table 2).**Maternal age** remained a significant continuous predictor (adjusted OR 1.29 per year; 95% CI 1.10–1.51; *p*=0.002), indicating that each additional year of age was associated with a 29% increase in the odds of PPH. This finding is consistent with reports that advanced maternal age may impair uterine contractility and increase bleeding risk due to underlying vascular changes and decreased myometrial responsiveness. **Gravidity** was also significant (OR 2.11 per increment; 95% CI 1.00–4.43; *p*=0.049). In practical terms, each additional pregnancy more than doubled the odds of PPH, reflecting the cumulative impact of **multiparity** (**each additional pregnancy more than one**) on the uterine musculature and placentation (e.g., placenta previa or accreta spectrum becoming more common with increasing parity). **Gestational age at delivery** had an inverse association with PPH risk (OR 0.64 per week; 95% CI 0.44–0.94; *p*=0.021). Thus, shorter gestations (earlier deliveries) were at higher risk of hemorrhage; for example, a delivery 4 weeks earlier in gestation (such as 33 weeks vs 37 weeks) corresponds to roughly a halving of odds for PPH, highlighting that the circumstances leading to preterm delivery (often severe APH from placental abruption or placenta previa) carry high hemorrhage risk. Finally, having received an **antepartum blood transfusion** was independently predictive of PPH (OR 2.44; 95% CI 1.02–5.84; *p*=0.045). This variable serves as a proxy for severity of the APH event; patients who required a transfusion prior to delivery were over twice as likely to hemorrhage postpartum, underscoring that severe ongoing bleeding or coagulopathy can extend into the postpartum period.

**Table 2.** Multivariable Logistic Regression Analysis for PPH.

**Predictor**	**β**	**SE**	**Adjusted OR**	**95% CI**	**P-value**
Maternal age (per 1 year)	0.256	0.081	1.29	1.10 – 1.51	0.002
Gravidity (per pregnancy)	0.746	0.378	2.11	1.00 – 4.43	0.049
Gestational age (per week)	−0.446	0.201	0.64	0.44 – 0.94	0.021
Antepartum transfusion (yes)	0.891	0.447	2.44	1.02 – 5.84	0.045

β = logistic regression coefficient; SE = standard error; OR = odds ratio; CI = confidence interval. Model fit: Nagelkerke R^2^ = 0.38; Hosmer-Lemeshow test p>0.05 (no lack of fit).

It is noteworthy that none of the other candidate variables (admission hemoglobin level, number of antenatal visits, or the timing intervals) remained significant in the adjusted analysis. In particular, the admission hemoglobin, despite many patients having anemia (mean ~9.5 g/dL), was not associated with PPH after controlling for other factors. This suggests that acute hemoglobin levels at the time of admission for APH did not predict subsequent PPH, whereas markers of bleeding severity (like transfusion need) did. It is important to distinguish this from pre-existing chronic anemia, which this study was not designed to isolate as a predictor. The final model had a Nagelkerke R^2^ of 0.38, indicating that approximately 38% of the variance in PPH outcome was explained by these four predictors.

### Model Performance and Internal Validation

**Figure 1.**
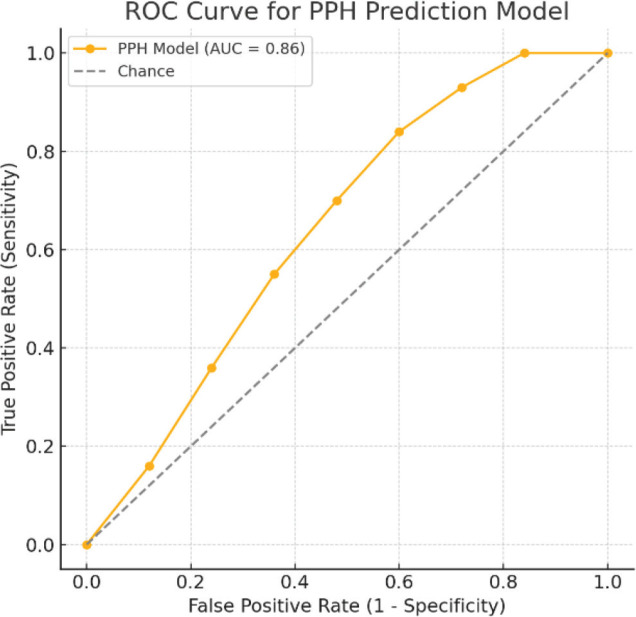
ROC Curve for the PPH Prediction Model. Demonstrates excellent model discrimination with AUC = 0.86.

**Figure 2.**
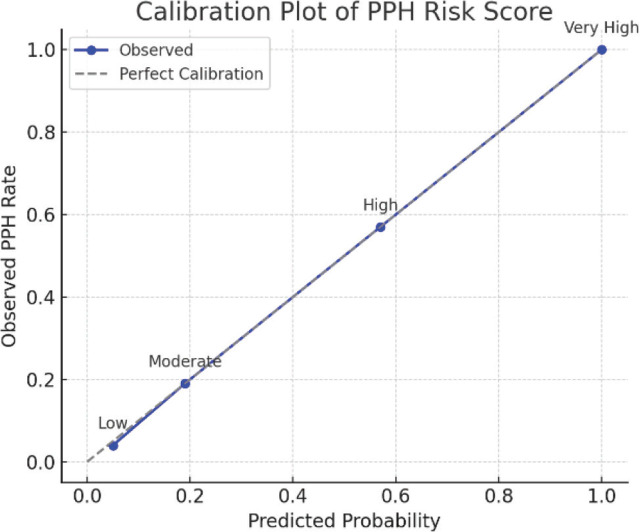
Calibration Plot. Shows agreement between observed and predicted PPH risk across four risk strata. The closer to the 45-degree line, the better the calibration.

The multivariable model demonstrated excellent discriminative ability. The area under the ROC curve (AUC) was 0.86 (95% CI 0.80–0.92) for predicting PPH, substantially exceeding the conventional threshold of 0.75 for a useful clinical model. Figure 1 illustrates the ROC curve for the model. The model’s calibration was also good: the calibration slope was 0.95, very close to the ideal of 1.0, and the calibration intercept was 0.02 (ideal 0) — indicating minimal systematic over- or under-prediction (Table 3, Figure 2). A Hosmer-Lemeshow goodness-of-fit test was non-significant (p>0.05), suggesting no evidence of lack of fit. The Nagelkerke R^2^ of 0.38 and Brier score of 0.18 further supported that the model had decent overall accuracy in this dataset.

**Table 3.** Model Performance and Internal Validation Metrics.

**Performance Metric**	**Value**	**95% CI**
**Discrimination**		
AUC (ROC curve) – apparent	0.86	0.80 – 0.92
AUC – optimism-corrected (bootstrap)	0.84	0.78 – 0.90
**Calibration**		
Calibration slope	0.95	–
Calibration intercept	0.02	–
Overall performance		
Nagelkerke R^2^	0.38	–
**Brier score**	0.18	–
**Internal validation (1000× bootstrap)**		
Optimism (AUC)	0.02	–
Uniform shrinkage factor	0.93	–

ROC = receiver operating characteristic; AUC = area under ROC curve. Optimism = difference between apparent and bootstrap performance.

Internal validation via bootstrap resampling (1,000 iterations) confirmed the model’s robustness. The optimism in the AUC was estimated at only 0.02, meaning the model’s performance did not substantially degrade on resampling. The optimism-corrected AUC was 0.84 (95% CI ~0.78–0.90), indicating that after adjusting for potential overfitting, the model would still be expected to achieve an AUC in the mid-0.80s on new data. In other words, there was minimal overfitting observed. The bootstrap procedure yielded a uniform shrinkage factor of 0.93, suggesting only a 7% reduction in coefficient magnitude would be needed to perfectly calibrate the model for overfitting — a sign of strong internal validity. Predictor inclusion frequencies were high (each of the four predictors appeared in >89% of bootstrap models), confirming that these variables were consistently selected and stable contributors to the model. These validation results increase confidence that the model’s predictions are reliable and not overly optimistic.

### Risk Score Development and Clinical Application

Using the four independent predictors, we constructed a Clinical Risk Score for PPH to facilitate practical use. The point allocation schema is detailed in Table 4. In summary, maternal age over 25 years contributed 1 point for every 4-year increment (capped at 2 points maximum); for example, a 33-year-old would receive 2 points. Gravidity contributed 1 point per pregnancy beyond the first (capped at 4 points); for instance, a woman who is Gravida 4 (three pregnancies beyond her first) would receive 3 points. Gestational age at delivery contributed up to 3 points on an inverse scale: deliveries at term (≥37 weeks) got 0 points (baseline risk), whereas near-term 33–36 weeks yielded 1 point, very preterm 28–32 weeks 2 points, and extremely preterm <28 weeks 3 points. For example, a delivery at 33 weeks carries 1 point. An antepartum transfusion before delivery contributed 2 points if it occurred (no points if no transfusion). These points were summed to produce a total risk score ranging from 0 to 11 in our population. Higher scores indicate greater predicted risk of PPH.

**Table 4.** Clinical Risk Score Components and Point Allocation.

**Predictor**	**Scoring Rule**	**Point Range**	**Example**
Maternal age (>25 years)	+1 point per 4-year increment above 25 (max 2 points)	0 – 2	Age 33 → 2 points
Gravidity (≥2 pregnancies)	+1 point per pregnancy above 1 (capped at 4)	0 – 4	Gravida 4 → 3 points
Gestational age (<37 wk)	28–32 wk = 2 pts; 33–36 wk = 1 pt; wk = 0 pts	0 – 2	33 weeks → 1 point
Antepartum transfusion	+2 points if present (any transfusion before delivery)	0 – 2	Yes (received) → 2 points
**Total possible score**	Sum of all component points (minimum 0, maximum 10)	**0 – 10**	–

**Figure 3.**
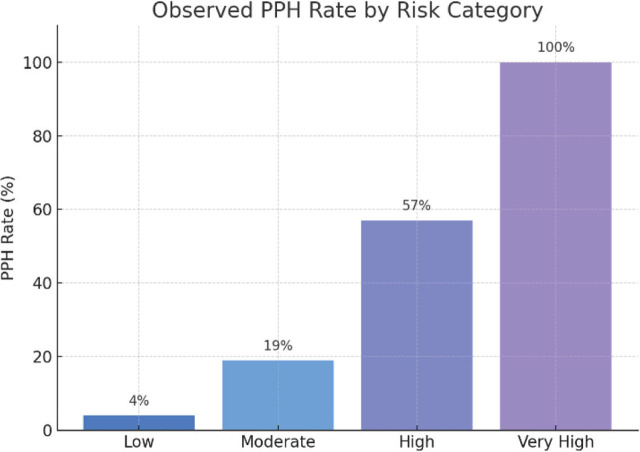
Bar Chart of PPH Rate by Risk Group. Illustrates the dramatic increase in observed PPH rate from Low (4%) to Very High (100%) risk category, validating the score’s clinical utility.

For ease of interpretation, we defined **four risk categories** based on the total score: **Low risk** (score 0–2), **Moderate risk** (3–5), **High risk** (6–8), and **Very high risk** (≥9 points). Each category corresponds to a stratum of PPH risk (Table 5). When we applied the score to our cohort, the distribution of patients across risk categories was as follows: 25% were low risk (n=25 with scores 0–2), 42% moderate (n=42 with scores 3–5), 28% high (n=28 with scores 6–8), and 5% very high risk (n=5 with scores ≥9). The observed PPH incidence rose progressively with each higher category, confirming the score’s ability to stratify risk (Figure 3). In the low-risk group, only 1 of 25 patients (4.0%) experienced PPH, a rate comparable to the general obstetric population’s baseline PPH risk. In the moderate-risk group, 8 of 42 (19.0%) had PPH — about five times the baseline risk. Among high-risk patients, 16 of 28 (57.1%) developed PPH, exceeding a 50% probability. Most strikingly, in the very high-risk group, all 5 of 5 patients (100%) had PPH. Thus, a score in the ≥9 range identified a subset of patients who invariably hemorrhaged, underscoring the need for immediate and aggressive management for anyone falling in this category. A chi-square test for trend showed a highly significant increase in PPH rates across the four risk strata (*p*<0.001 for trend).

**Table 5.** Risk Stratification by Clinical Risk Score.

**Risk Category**	**Score Range**	**Patients (n=100)**	**PPH Cases (n)**	**PPH Rate % (95% CI)**	**Predicted Risk Range %**
Low	0 – 2	25 (25.0%)	1	4.0% (0.1 – 20.4)	~3 – 10%
Moderate	3 – 5	42 (42.0%)	8	19.0% (8.6 – 34.1)	~15 – 30%
High	6 – 8	28 (28.0%)	16	57.1% (37.2 – 75.5)	~40 – 64%
Very High	≥ 9	5 (5.0%)	5	100.0% (47.8 – 100.0)	≥ 75%
**Tota**l	0 – 10	100 (100%)	30	30.0% (21.2 – 40.1)	–

PPH = postpartum hemorrhage; CI = confidence interval. Trend test across risk categories: p<0.001 (increasing PPH rate with higher score category).

We also evaluated the diagnostic performance of various score thresholds for predicting PPH (Table 6). Using any nonzero score (threshold ≥1) as a positive test resulted in a sensitivity of 100% (95% CI 88.4–100%), meaning the score identified all 30 PPH cases in our cohort (no PPH case had a total score of 0). However, this low cutoff yielded a specificity of only 35.7%, as many patients without PPH still had at least 1 point. Accordingly, the positive predictive value (PPV) at ≥1 was only 36.6%, reflecting a high false-positive rate, while the negative predictive value (NPV) was 100% (no false negatives). At a more clinically useful threshold of ≥3 (dichotomizing moderate-risk and above vs. low-risk), the sensitivity was 96.7% and specificity 77.1%. In other words, using a score ≥3 to “flag” high-risk patients would capture 29 out of 30 PPH cases, while correctly identifying about 77% of those who would not hemorrhage. This threshold offers a good balance: it ensures nearly all true PPH cases are predicted (only 1 case would be missed) and substantially reduces false alarms compared to using ≥1. The PPV at ≥3 was 65.9%, and NPV 98.1%. Using a higher threshold of ≥6 (to identify only the high and very high-risk patients) yields lower sensitivity (70.0%) but higher specificity (88.6%). A score ≥6 correctly flagged 21 of 30 PPH cases and correctly ruled out ~89% of non-cases; PPV was 75.0% and NPV 86.1%. Finally, the extreme threshold of ≥9 (identifying only the very high risk group) had sensitivity 16.7% (it picked up only 5 of 30 PPH cases, as expected) but specificity 100%. Thus, a score in this top category virtually guarantees PPH (PPV 100%) but will only apply to a small fraction of patients. These analyses allow clinicians to choose cut-points based on the context: for screening purposes a lower threshold (≥3) may be preferred to maximize sensitivity, whereas for allocating scarce resources (e.g., blood products, transfer to ICU), a higher threshold (≥6 or ≥9) could be used to focus on those most likely to benefit from intervention.

**Table 6.** Predictive Performance of Risk Score at Various Thresholds.

**Threshold (Score ≥)**	**Sensitivity (95% CI)**	**Specificity (95% CI)**	**PPV (95% CI)**	**NPV (95% CI)**	**LR+**	**LR−**	**Accuracy**
≥ 1 point	100.0% (88.4 – 100.0)	35.7% (24.6 – 48.1)	36.6% (27.0 – 47.2)	100.0% (86.3 – 100.0)	1.56	0.00	56.0%
≥ 3 points	96.7% (82.8 – 99.9)	77.1% (65.6 – 86.3)	65.9% (50.1 – 79.5)	98.1% (89.7 – 100.0)	4.22	0.04	84.0%
≥ 6 points	70.0% (50.6 – 85.3)	88.6% (78.7 – 94.9)	75.0% (55.1 – 89.3)	86.1% (75.9 – 93.4)	6.13	0.34	84.0%
≥ 9 points	16.7% (5.6 – 34.7)	100.0% (94.9 – 100.0)	100.0% (47.8 – 100.0)	76.0% (66.4 – 84.2)	∞	0.83	80.0%

PPV = positive predictive value; NPV = negative predictive value; LR+ = positive likelihood ratio; LR− = negative likelihood ratio. All values are percentages except likelihood ratios. Percentages are followed by 95% confidence intervals in parentheses.

In summary, the point-based risk score stratified APH patients into meaningful risk tiers and demonstrated strong diagnostic utility. Its performance compares favorably with existing risk assessment tools, as discussed below.

### Comparative Performance Analysis

Compared to existing PPH risk assessment tools, our model shows improved performance. For example, the California Maternal Quality Care Collaborative (CMQCC) hemorrhage risk index — a risk stratification tool widely used in obstetric practice — reportedly achieves an AUC in the range of only ~0.66–0.69 in validation studies. Meanwhile, contemporary machine-learning models for general obstetric populations have published AUCs ranging from ~0.72 up to 0.81. In contrast, our APH-specific model attained an AUC of 0.86, representing a notable improvement in predictive accuracy. This superior discrimination likely reflects our focus on a high-risk subpopulation (women with APH) and the inclusion of clinically relevant variables specific to APH pathophysiology. We believe that a targeted model can outperform more generic PPH prediction models when applied to the APH context.

## Discussion

We developed and internally validated a novel clinical risk score to predict postpartum hemorrhage in women with antepartum hemorrhage. The model, based on four easily measured predictors — maternal age, gravidity, gestational age, and antepartum transfusion requirement — demonstrated excellent discriminative ability (AUC 0.86) and good calibration in our cohort. The point-based score stratified patients into low, moderate, high, and very high risk groups with observed PPH rates of approximately 4%, 19%, 57%, and 100%, respectively (Table 5). This clear risk gradient suggests the score has practical clinical utility for identifying women with APH who are at greatest risk of significant hemorrhage. Notably, even the moderate-risk group (score ≥3) had a nearly 20% PPH rate — several-fold higher than baseline — which underscores that any woman with an APH accumulating a few risk factors warrants careful management. At the highest scores, the risk was virtually certain, highlighting a subgroup that may benefit from maximal preparedness (e.g., arranging blood products, involving senior clinicians, and readying uterotonic/uterine surgical interventions in advance).

The model’s performance is particularly encouraging when compared to previously published PPH prediction efforts. Ende *et al.* recently developed an automated real-time PPH prediction model in a general obstetric population that achieved an AUC of 0.81 [7]. In another study, Venkatesh *et al.* reported AUCs of 0.67–0.72 when externally validating various PPH risk models on electronic health record data [9,10]. Our model’s AUC of 0.86 is higher than these, suggesting a meaningful improvement in predictive accuracy. This boost in performance likely stems from our focus on an APH cohort — a group with distinctive risk factors and pathophysiology — combined with rigorous development methods. By tailoring the model to women with APH, we captured risk indicators (like transfusion need, gestational age at delivery) that are especially pertinent in this scenario but might not feature in general population models. Additionally, our use of bootstrap validation and careful calibration may contribute to more reliable performance estimates.

The identification of gestational age as a *protective* factor (i.e., shorter gestation = higher risk of PPH) provides important insight into APH-related hemorrhage. In general obstetric populations, delivering at term or post-term can sometimes increase hemorrhage risk due to larger placental size and uterine atony in prolonged pregnancies [11,12]. However, in the context of APH, our finding that earlier deliveries are associated with higher PPH risk likely reflects the fact that many preterm deliveries in APH are precipitated by severe placental pathologies (such as a major placental abruption or placenta previa with heavy bleeding). These conditions cause hemorrhage both antepartum and postpartum through mechanisms like defective placental implantation, coagulopathy, and uterine atony from abruptio placentae. In essence, the gestational age effect in our model does not imply a direct biological cause but rather serves as a clinical proxy for the severity and underlying etiology of APH (e.g., severe placental abruption necessitating early delivery): an APH severe enough to cause a very preterm delivery inherently carries a high likelihood of PPH. This contrasts with the general obstetric setting where gestational age by itself is not usually a dominant predictor of hemorrhage.

The associations of advanced maternal age and higher gravidity/parity with PPH risk align with well-established obstetric literature. Advanced maternal age has been associated with an increased risk of atonic PPH, possibly due to age-related myometrial changes or higher incidence of placenta previa and accreta in older gravidas [9,10]. Likewise, multiparity is known to increase risks of abnormal placentation (e.g., placenta previa/accreta) and uterine atony, as well as to reflect the cumulative toll of repeated pregnancies on uterine contractility. Our results reinforce these concepts in the specific subset of women with APH, confirming that maternal age and gravidity remain relevant risk factors. Liu *et al.* (2022) and Watanabe *et al.* (2023) similarly identified maternal age and parity as significant predictors of PPH using both traditional statistical models and machine learning approaches [11,12], lending support to the generalizability of these risk factors.

Our model also incorporated antepartum transfusion as a predictor, which is somewhat unique. This variable effectively captured the severity of the APH event — functioning as a summary measure of the bleeding intensity and coagulopathy prior to delivery. By including it, the model accounts for the “state” of the patient heading into delivery (e.g., depleted reserves or ongoing bleeding). Interestingly, recent studies have emphasized combining antepartum and intrapartum factors for PPH prediction. Our use of a pre-delivery transfusion as a predictor echoes that principle, integrating an element of the clinical course of APH (not just baseline characteristics). This dynamic risk assessment throughout the peripartum period can improve prediction, as demonstrated by investigators in different settings [13]. Importantly, this factor is readily available to clinicians and is essentially a red flag that the patient has already declared herself high-risk by virtue of needing blood before delivery.

Methodologically, this study has several strengths. We adhered to TRIPOD guidelines in model development and reporting [7,8]. We used *bootstrap internal validation*, which is more efficient and informative than a simple training/test split given our sample size. The bootstrap validation confirmed minimal overfitting (optimism ~0.02) and yielded a high shrinkage factor (0.93), supporting the stability of the model. We also evaluated not just discrimination but calibration and decision-level utility (via risk stratification and predictive values), providing a comprehensive picture of performance that meets contemporary standards for prediction model evaluation [7,8]. By focusing on quantitative blood loss measurement where possible and a clear outcome definition, we improved outcome ascertainment accuracy, which is critical for model reliability. Studies have consistently shown that quantitative blood loss assessment is superior to visual estimation, particularly at higher volumes relevant to PPH [14,15], so our approach likely reduced outcome misclassification.

From a clinical standpoint, the point-based risk score we derived is a key translational aspect of this work. It condenses the logistic model into a simple tool that can be used bedside without calculators. Each variable in the score is routinely obtained in clinical practice, and the scoring system (Table 4) is straightforward. We deliberately kept the number of predictors small (four) to favor ease of use and to minimize overfitting given our sample size. This aligns with the critical need for simple, interpretable risk assessment tools highlighted in recent reviews and guidelines. For instance, the California Maternal Quality Care Collaborative (CMQCC) obstetric hemorrhage toolkit emphasizes early risk stratification on admission using basic obstetric history and clinical factors [16]. Our score could be integrated into such workflows: for a woman admitted with APH, one could quickly tally her points (e.g., age, gravidity, gestational age, transfusion status) and assign a risk category. This risk stratification can guide clinical decisions such as where the delivery should occur (e.g., ensuring availability of blood bank and higher-level care for high-risk cases), alerting the hemorrhage response team, preparing uterotonics, or arranging for uterine artery embolization prophylactically in placenta previa cases.

Identifying the subset of very high-risk patients (score ≥9) is particularly valuable. In our data, this group had 100% PPH incidence. While this will need confirmation in larger samples, it suggests that when multiple risk factors converge (e.g., an older, multiparous woman with a very preterm APH who already required transfusion), clinicians should anticipate a hemorrhage as a near certainty. For such patients, maximal preparedness is warranted: for example, delivering in an operating theater, having blood products cross-matched and immediately available, and assembling a multidisciplinary team (obstetricians, anesthesiologists, interventional radiology if available) *before* delivery. Recent international guidelines on obstetric hemorrhage management call for individualized care plans based on risk assessment [17] — our score provides an objective way to define “very high risk” and could thereby trigger these intensive preventive measures.

**Global health implications:** The simplicity of the score (using variables like age, gravidity, etc.) makes it especially relevant for resource-limited settings, where APH-related maternal mortality is highest. In many low-resource hospitals, advanced laboratory tests or massive transfusion protocols are not readily available, so early identification of high-risk women is crucial for timely referral and intervention. Our model relies on basic clinical information and thus could be applied in settings without sophisticated infrastructure. Studies from Kenya and Ethiopia have shown that even in low-resource environments, prediction tools based on simple clinical parameters can significantly stratify PPH risk [2]. The World Health Organization’s strategies toward ending preventable maternal mortality by 2030 emphasize the development of practical risk assessment tools that can be used globally [18]. The performance of our model using routine antenatal and intrapartum data supports its potential for widespread implementation, particularly in settings where more complex predictors (like detailed lab results or imaging) are impractical. By improving early risk recognition, such a tool could help optimize the use of limited resources (e.g., prioritizing blood units and uterotonics for those most in need) and ultimately reduce preventable PPH deaths.

There is also a growing recognition of racial and ethnic disparities in PPH outcomes, even in high-resource countries. These disparities are thought to stem from a combination of biological, systemic, and care-related factors. An objective risk score like ours could support more equitable care by ensuring that all patients are assessed with the same criteria, potentially mitigating subjective biases in identifying who is “high-risk.” Validated tools can prompt early intervention for anyone flagged as high-risk, which may help narrow outcome gaps. Our model is founded primarily on physiological and historical risk factors rather than socioeconomic or provider impression, providing a standardized approach to risk stratification. That said, it will be important to validate it in diverse populations to confirm its accuracy across different racial/ethnic groups and settings. External validations could also explore whether adding or modifying predictors is needed to maintain performance in other contexts.

**Limitations:** Several limitations of our study warrant acknowledgment. First, this was a single-center study with a modest sample size (N=100, with 30 PPH events). Although this meets traditional events-per-variable guidelines for model development, the sample size limited our power to detect smaller effect sizes and precluded inclusion of more predictors. The model may not capture rare predictors of PPH, and some potentially important variables (e.g., clinically estimated blood loss of APH, fibrinogen levels) were not available in our dataset. Second, we performed only internal validation. The model’s generalizability to other hospitals or populations is unproven until external validation is conducted. Because obstetric practices and patient characteristics vary, a model derived in one setting might perform differently elsewhere. We thus emphasize that the score should be validated in independent cohorts (both within and outside of India) before broad clinical adoption. Third, the inclusion of transfusion in the outcome definition could introduce incorporation bias for the ‘antepartum transfusion’ predictor. While antepartum transfusion is a distinct event occurring before delivery, both may reflect the same underlying hemorrhagic diathesis. This variable should be viewed as a marker of disease severity rather than an independent baseline risk factor. However, we note that, by definition, all postpartum transfusions would count as PPH outcomes; the predictor in our model was *antepartum* transfusion (before delivery), which is distinct and thus did not logically guarantee the outcome. Lastly, we did not have an external comparison group or an impact analysis — it remains to be seen how use of this risk score might change clinical practice or improve outcomes. Future studies should assess whether implementing the score prospectively leads to better preparation and reductions in PPH severity.

The inclusion of antepartum transfusion may introduce incorporation bias. This variable should be viewed as a marker of disease severity rather than an independent baseline risk factor.

## Conclusion

This study presents a novel, internally validated clinical risk score (pending external validation) for predicting postpartum hemorrhage in women with antepartum hemorrhage, demonstrating excellent discriminative ability and practical clinical utility. The four-variable model (maternal age, gravidity, gestational age, and antepartum transfusion) provides a simple yet accurate tool for early risk identification, potentially enabling targeted interventions to reduce maternal morbidity and mortality. Its superior performance compared to existing tools, combined with its ease of use and strong internal validation, position it as a promising candidate for clinical implementation once external validation is achieved. In particular, the ability to identify a very high-risk subset of APH patients (with near-certain hemorrhage) offers valuable opportunities for proactive management, resource allocation, and multidisciplinary planning.

Future research should focus on external validation of this risk score in diverse populations and settings to confirm its generalizability. Additionally, integrating the score into clinical workflows (for example, as part of obstetric early warning systems or hemorrhage protocols) and evaluating its impact on clinical decision-making and patient outcomes will be important next steps. The model’s foundation in readily available clinical variables and its excellent performance characteristics suggest substantial potential for improving maternal care globally, particularly in resource-limited settings where the burden of APH-related PPH is highest and where simple predictive tools can have the greatest impact.
